# Higher fasting triglyceride predicts higher risks of diabetes mortality in US adults

**DOI:** 10.1186/s12944-021-01614-6

**Published:** 2021-12-20

**Authors:** Yutang Wang

**Affiliations:** Discipline of Life Sciences, School of Science, Psychology and Sport, Federation University Australia, University Drive, Mt Helen, VIC 3350 Australia

**Keywords:** Triglyceride, Diabetes, Mortality, Biomarker, Fasting, Association, Risk factor

## Abstract

**Background:**

It is unknown whether higher triglyceride results in higher mortality from diabetes, i.e., diabetes mortality. This study aimed to investigate the association of fasting triglyceride with diabetes mortality.

**Methods:**

This study included 26,582 US adults from the National Health and Nutrition Examination Surveys from 1988 to 2014. Diabetes mortality outcomes were ascertained by linkage to the National Death Index records. Cox proportional hazards models were used to estimate hazard ratios (HRs) and 95% confidence intervals (CIs) of triglyceride for diabetes mortality.

**Results:**

Higher levels of fasting triglyceride were associated with higher levels of glucose, glycated hemoglobin, insulin, and homeostatic model assessment for insulin resistance at baseline. A 1-natural-log-unit increase in triglyceride (e.g., from 70 to 190 mg/dL) was associated with a 115% higher multivariate-adjusted risk of diabetes diagnosis (odds ratio, 2.15; 95% CI, 2.00–2.33). During 319,758 person-years of follow-up with a mean follow-up of 12.0 years, 582 diabetes deaths were documented. Compared with people with triglyceride in the lowest quintile, people with triglyceride in the highest quintile had an 85% higher risk of diabetes mortality (HR, 1.85; 95% CI, 1.25–2.73). A 1-natural-log-unit increase in triglyceride was associated with a 40% higher multivariate-adjusted risk of diabetes mortality. The positive association between triglyceride and diabetes mortality was also presented in sub-cohorts of participants with or without diabetes.

**Conclusions:**

This study demonstrated that higher fasting triglyceride was associated with a higher diabetes mortality risk.

**Supplementary Information:**

The online version contains supplementary material available at 10.1186/s12944-021-01614-6.

## Introduction

About 34.1 million adults—or 13.0% of all US adults—have diabetes, the seventh leading cause of death in the United States with a crude death rate of 25.7 per 100,000 persons per year [1]. The total direct and indirect estimated costs of diagnosed diabetes in the United States in 2017 alone were $327 billion [1]. Therefore, it is of great importance to identify modifiable factors to decrease diabetes incidence and diabetes-induced death, i.e., diabetes mortality.

Epidemiological studies frequently show that circulating triglyceride is higher in people with diabetes than that in those without diabetes [2, 3]. Higher baseline triglyceride was reported to be associated with new-onset of diabetes in various populations such as Chinese [4], Japanese [5], and Americans [6]. These studies support the notion that higher triglyceride could lead to diabetes.

However, it is unknown whether higher circulating triglyceride leads to higher diabetes mortality. This study aimed to investigate whether higher fasting triglyceride at baseline predicted diabetes mortality using US adults who attended the National Health and Nutrition Examination Survey (NHANES) from 1988 to 2014.

## Methods

### Study participants

This study included participants from NHANES III (1988–1994) and the subsequent eight cycles of NHANES from 1999 to 2014. The inclusion criteria included age of ≥20 years and presence of the fasting triglyceride data. This resulted in a cohort of 27,067 participants. The following participants were excluded: those without a follow-up time or with a follow-up time of 0 month (*N* = 31), and those who did not have data on the following parameters: plasma glucose (*N* = 57), blood glycated hemoglobin (HbA_1c_, *N* = 70), serum insulin (*N* = 239), physical activity (*N* = 11), and education status (*N* = 77). Therefore, a total of 26,582 participants were included in the final analysis.

### Fasting triglyceride

Fasting status was defined as a fasting time between 8.0 and 23.9 h. The concentration of fasting triglyceride in the serum was retrieved from the NHANES website. Triglyceride was measured enzymatically by using a series of coupled reactions in which triglyceride was hydrolyzed to produce glycerol [7]. Glycerol was then phosphorylated and oxidized to produce H_2_O_2_, and the latter, in the presence of peroxidase, produced a color product that was measured spectrophotometrically at the wavelength of 500 nm. Triglyceride was treated as a continuous variable (natural log-transformed) or a categorical variable (quintiles) in the analysis.

### Diabetes-related markers and definition of diabetes

The following diabetes-related markers were retrieved from the NHANES website: plasma glucose, HbA_1c_ in the whole blood, and serum insulin. Homeostatic model assessment (HOMA) for insulin resistance was calculated using the following formula [8]: (serum insulin in μU/mL X plasma glucose in mmol/L)/22.5.

Diabetes was defined as fasting plasma glucose ≥126 mg/dL, or HbA_1c_ ≥ 6.5%, or taking hypoglycemic drugs, or self-reported diagnosis [9].

### Diabetes mortality

Data on diabetes-caused mortality were directly retrieved from NHANES-linked mortality files. To evaluate mortality status and the cause of death, the National Center for Health Statistics conducted probabilistic matching to link the NHANES data with death certificate records from the National Death Index records. The NHANES-linked mortality files used the Underlying Cause of Death 113 (UCOD_113) code to recode all deaths according to the International Classification of Diseases, 9th Revision (ICD-9) or the International Classification of Diseases, 10th Revision (ICD–10). Diabetes mortality was defined as diabetes being listed as an underlying cause of death. Follow-up time was defined as the time (in months) from the time when the blood was drawn at the Mobile Examination Center until death, or until the end of follow-up (i.e., December 31, 2015), whichever occurred first.

### Covariates

Confounding covariates included age (continuous) [2], sex (male or female) [10], ethnicity (non-Hispanic white, non-Hispanic black, Mexican-American, or other) [11], obesity (underweight, normal, overweight, obese, or unknown) [3, 12], education (< high school, high school, or > high school) [2], poverty-income ratio (< 130, 130–349%, ≥ 350%, or unknown) [10, 13], and survey periods (1988–1991, 1991–1994, 1999–2000, 2001–2002, 2003–2004, 2005–2006, 2007–2008, 2009–2010, 2011–2012, or 2013–2014). NHANES III was conducted in two stages, i.e., from 1988 to 1991 and then from 1991 to 1994, and the subsequent cycles of NHANES were conducted once every 2 years. Lifestyle confounders included physical activity (inactive, insufficiently active, or active) [14, 15], alcohol consumption (never, < 1 drink per week, 1–6 drinks per week, ≥ 7 drinks per week, or unknown) [16, 17], and smoking status (past smoker, current smoker, or unknown) [18]. Physical activity was defined as previously reported [15]. In brief, the inactive group was defined as those with no reported moderate-to-vigorous leisure-time physical activity; the active group was defined as those who had ≥3 times per week of vigorous leisure-time physical activities, or a combination of 2 vigorous and 2 moderate leisure-time physical activities per week, or ≥ 5 times per week of any type of leisure-time physical activities (moderate or vigorous or a combination); and insufficiently active group was defined as those who were not inactive and did not meet the criteria for the active group [15]. Clinical confounders included hypercholesterolemia (yes or no) [19], diabetes (yes or no), hypertension (yes, no, or unknown) [2], and family history of diabetes (yes, no, or unknown) [2]. Hypercholesterolemia was defined as total cholesterol ≥240 mg/dL or self-reported diagnosis of hypercholesterolemia [20]. Hypertension was defined as systolic blood pressure ≥ 140 mmHg or diastolic blood pressure ≥ 90 mmHg or prior diagnosis or treatment of hypertension [21].

### Statistical analyses

Data were presented as mean and standard deviation for continuous variables or percentages for categorical variables. Difference in age was analyzed using Student’s t-test and differences in non-normally distributed continuous variables (triglyceride, glucose, HbA_1c_, insulin, HOMA for insulin resistance, and C-reactive protein) were analyzed using the Mann-Whitney U test between those with or without diabetes. Differences among categorical variables were analyzed using Pearson’s chi-square test. Associations of triglyceride with diabetes-related markers (glucose, HbA_1c_, insulin, and HOMA for insulin resistance) were assessed using the least-squares regression [22], with or without adjustment for confounding factors including age, sex, ethnicity, obesity, education, income, lifestyle factors (physical activity, alcohol consumption, and smoking status), survey period, and clinical confounders (hypercholesterolemia, hypertension, diabetes, and family history of diabetes). Triglyceride, glucose, HbA_1c_, insulin, and HOMA for insulin resistance were natural log-transformed to improve the data distribution before being put into the models. Binary logistic regression was used to assess the association of triglyceride with diabetes diagnosis, with or without adjustment for the above confounders [23]. Cox proportional hazards models were used to calculate hazard ratios (HRs) and 95% confidence intervals (CIs) of triglyceride for diabetes mortality. Sub-analyses were conducted in sub-cohorts of participants with or without diabetes.

Sensitivity analyses were conducted by further adjustment for baseline glucose, or high-density lipoprotein (HDL) cholesterol and low-density lipoprotein (LDL) cholesterol, as higher levels of glucose [11], lower levels of HDL cholesterol [24], and higher levels of LDL cholesterol [25, 26] were associated with new-onset of diabetes. Sensitivity analyses were also conducted by further adjustment for C-reactive protein [27] or the use of lipid-lowering drugs. In addition, sensitivity analyses were conducted when 2-h post-load glucose was considered as a criterium for diabetes diagnosis or when diabetes mortality was defined as diabetes being listed as the leading cause of death.

The null hypothesis was rejected for two-sided values of *P* <  0.05. All analyses were performed using SPSS version 27.0 (IBM SPSS Statistics for Windows, Armonk, NY, IBM Corporation).

## Results

### General characteristics

This study included 26,582 (22,909 without diabetes and 3673 with diabetes) US adults with a mean (SD) age of 49 (19) years. Baseline characteristics of the participants are displayed in Table 1.

**Table 1:** Baseline characteristics of 26,582 US adults

	Participants without diabetes	Participants with diabetes	All participants	*P* value
Sample size	22,909	3673	26,582	NA
Age, y, mean (SD)	47 (18)	61 (14)	49 (19)	< 0.001
Sex (male), %	47.4	50.9	47.9	< 0.001
Ethnicity, %				< 0.001
Non-Hispanic white	46.2	38.8	45.2	
Non-Hispanic black	21.3	24.8	21.8	
Mexican American	21.8	24.1	22.1	
Other	10.8	12.2	11.0	
Triglyceride, mg/dL, mean (SD)	130 (97)	186 (192)	137 (117)	< 0.001
PG, mg/dL, mean (SD)	96 (10)	156 (63)	104 (33)	< 0.001
HbA_1c_, %, mean (SD)	5.3 (0.4)	7.3 (1.8)	5.6 (1.0)	< 0.001
Insulin, μU/mL, mean (SD)	11.1 (8.4)	18.5 (22.8)	12.1 (11.8)	< 0.001
HOMA-IR, mean (SD)	2.7 (2.2)	7.2 (9.8)	3.3 (4.5)	< 0.001
CRP, mg/dL, mean (SD)	0.44 (0.79)	0.65 (1.01)	0.47 (0.83)	< 0.001
Obesity, %				< 0.001
Underweight	1.8	0.7	1.6	
Normal	34.5	14.6	31.7	
Overweight	34.8	31.3	34.3	
Obese	28.0	51.4	31.3	
Unknown	0.9	2.0	1.1	
Poverty-income ratio, %				< 0.001
< 130%	27.8	33.5	28.6	
130–349%	36.8	38.0	37.0	
≥ 350%	27.2	18.9	26.1	
Unknown	8.1	9.5	8.3	
Education, %				< 0.001
< High School	30.5	43.9	32.3	
High School	26.0	24.1	25.7	
> High School	43.5	32.0	41.9	
Physical activity, %				< 0.001
Inactive	27.5	19.1	26.4	
Insufficiently active	38.3	30.3	37.2	
Active	34.2	50.6	36.5	
Alcohol consumption, %				< 0.001
0 drink/week	16.3	27.3	17.8	
< 1 drink/week	22.8	20.3	22.5	
1–6 drinks/week	21.5	11.6	20.2	
≥ 7 drinks/week	13.5	8.9	12.8	
Unknown	25.8	31.9	26.7	
Smoking status, %				< 0.001
Past smoker	23.3	17.5	22.5	
Current smoker	23.6	35.0	25.2	
Unknown	53.1	47.5	52.3	
Hypercholesterolemia, %	32.1	53.6	35.1	< 0.001
Hypertension, %	34.3	69.5	39.1	< 0.001
Diabetes, %	0	100	13.8	NA
Family history of diabetes, %	40.6	61.9	43.5	< 0.001
Use of lipid-lowering drugs, %	8.3	30.2	11.3	< 0.001

Abbreviations: *CRP* C-reactive protein, *HbA*_*1c*_ glycated hemoglobin, *HOMA-IR* homeostatic model assessment for insulin resistance, *NA* not applicable, *PG* plasma glucose, *SD* standard deviation

### Association of circulating triglyceride with diabetes-related markers

Higher triglyceride was independently associated with higher plasma glucose, higher HbA_1c_, higher serum insulin, and higher HOMA for insulin resistance in the whole cohort and sub-cohorts of participants with or without diabetes (Table 2).

**Table 2:** Association of triglyceride (independent variable) ^a^ with diabetes markers (dependent variables) ^a^ in 26,582 adults

	Model 1		Model 2		Model 3		Model 4	
	β	*P* value	β	*P* value	β	*P* value	β	*P* value
All participants (*N* = 26,582)								
Plasma glucose	0.275	< 0.001	0.226	< 0.001	0.193	< 0.001	0.114	< 0.001
Blood HbA_1c_	0.223	< 0.001	0.192	< 0.001	0.159	< 0.001	0.074	< 0.001
Serum insulin	0.367	< 0.001	0.395	< 0.001	0.268	< 0.001	0.258	< 0.001
HOMA-IR	0.401	< 0.001	0.411	< 0.001	0.290	< 0.001	0.259	< 0.001
Participants without diabetes (*N* = 22,909)								
Plasma glucose	0.200	< 0.001	0.119	< 0.001	0.077	< 0.001	0.074	< 0.001
Blood HbA_1c_	0.119	< 0.001	0.086	< 0.001	0.050	< 0.001	0.035	< 0.001
Serum insulin	0.358	< 0.001	0.393	< 0.001	0.267	< 0.001	0.268	< 0.001
HOMA-IR	0.366	< 0.001	0.386	< 0.001	0.262	< 0.001	0.261	< 0.001
Participants with diabetes (*N* = 3673)								
Plasma glucose	0.286	< 0.001	0.284	< 0.001	0.288	< 0.001	0.292	< 0.001
Blood HbA_1c_	0.200	< 0.001	0.215	< 0.001	0.213	< 0.001	0.204	< 0.001
Serum insulin	0.250	< 0.001	0.250	< 0.001	0.209	< 0.001	0.216	< 0.001
HOMA-IR	0.342	< 0.001	0.341	< 0.001	0.306	< 0.001	0.315	< 0.001

Abbreviations: *HbA*_*1c*_ glycated hemoglobin, *HOMA-IR* homeostatic model assessment for insulin resistance

^a^Triglyceride, plasma glucose, blood HbA_1c_, serum insulin, and HOMA-IR, were natural log-transformed. Model 1: unadjusted; Model 2: adjusted for age, sex, and ethnicity; Model 3: adjusted for all the factors in Model 2 plus obesity, poverty-income ratio, education, physical activity, alcohol consumption, smoking status, and survey period; Model 4: adjusted for all the factors in Model 3 plus hypercholesterolemia, hypertension, diabetes, and family history of diabetes

### Association of circulating triglyceride with diabetes diagnosis

A 1-natural-log-unit increase in triglyceride (e.g., from 70 to 190 mg/dL) was associated with a higher multivariate-adjusted risk of diabetes diagnosis (odds ratio, 2.15; 95% CI, 2.00–2.33; *P* <  0.001; Table 3).

**Table 3:** Natural log-transformed triglyceride and risk for diabetes diagnosis among 26,582 adults

	Odds ratio	95% CI	*P* value
Model 1	2.52	2.38–2.68	< 0.001
Model 2	2.64	2.47–2.82	< 0.001
Model 3	2.38	2.22–2.56	< 0.001
Model 4	2.15	2.00–2.33	< 0.001

Abbreviations: *CI* confidence interval

Model 1: unadjusted; Model 2: adjusted for age, sex, and ethnicity; Model 3: adjusted for all the factors in Model 2 plus obesity, poverty-income ratio, education, physical activity, alcohol consumption, smoking status, and survey period; Model 4: adjusted for all the factors in Model 3 plus hypercholesterolemia, hypertension, and family history of diabetes

### Association of circulating triglyceride with diabetes mortality

During 319,758 person-years of follow-up with a mean follow-up of 12.0 years, 582 participants died from diabetes. A 1-natural-log-unit increase in triglyceride was associated with a 40, 32, and 45% higher multivariate-adjusted risk of diabetes mortality in the whole cohort, and sub-cohorts of participants with or without diabetes, respectively (Table 4).

**Table 4:** Natural log-transformed triglyceride and risk for diabetes mortality among 26,582 adults

	All participants (*N* = 26,582)			Participants without diabetes (*N* = 22,909)			Participants with diabetes (*N* = 3673)		
	HR	95% CI	*P* value	HR	95% CI	*P* value	HR	95% CI	*P* value
Model 1	2.62	2.33–2.94	< 0.001	2.22	1.77–2.78	< 0.001	1.32	1.14–1.54	< 0.001
Model 2	2.35	2.05–2.68	< 0.001	1.77	1.37–2.28	< 0.001	1.47	1.24–1.75	< 0.001
Model 3	2.12	1.84–2.45	< 0.001	1.51	1.15–1.99	0.003	1.39	1.17–1.67	< 0.001
Model 4	1.40	1.20–1.64	< 0.001	1.45	1.09–1.92	0.011	1.32	1.09–1.58	0.004

Abbreviations: *CI* confidence interval, *HR* hazard ratio

Model 1: unadjusted; Model 2: adjusted for age, sex, and ethnicity; Model 3: adjusted for all the factors in Model 2 plus obesity, poverty-income ratio, education, physical activity, alcohol consumption, smoking status, and survey period; Model 4: adjusted for all the factors in Model 3 plus hypercholesterolemia, hypertension, diabetes, and family history of diabetes

Compared with people with triglyceride in the lowest quintile, people with triglyceride in the highest quintile had a higher risk of diabetes mortality in the whole cohort (HR, 1.85; 95% CI, 1.25–2.73; *P* = 0.002), and in the sub-cohorts of participants with (HR, 1.68; 95% CI, 1.15–2.45; *P* = 0.007) or without diabetes (HR, 2.80; 95% CI, 1.39–5.63; *P* = 0.004; Table 5).

**Table 5:** Triglyceride in quintiles and risk for diabetes mortality among 26,582 adults

Triglyceride in quintiles	No. of participants	No. of deaths	HR ^a^ (95% CI)	*P* value
All participants (*N* = 26,582)				
Q 1 ≤ 70 mg/dL	5225	32	1 [reference]	NA
Q2 71–95 mg/dL	5321	58	1.10 (072–1.71)	0.656
Q3 96–126 mg/dL	5296	109	1.42 (0.95–2.13)	0.086
Q4 127–180 mg/dL	5422	141	1.35 (0.91–2.02)	0.138
Q5 ≥ 181 mg/dL	5318	242	1.85 (1.25–2.73)	0.002
Participants without diabetes (*N* = 22,909)				
Q 1 ≤ 68 mg/dL	4535	10	1 [reference]	NA
Q2 69–92 mg/dL	4626	28	1.88 (0.91–3.89)	0.089
Q3 93–121 mg/dL	4540	37	2.06 (1.01–4.19)	0.046
Q4 122–170 mg/dL	4601	47	2.06 (1.02–4.17)	0.044
Q5 ≥ 171 mg/dL	4607	70	2.80 (1.39–5.63)	0.004
Participants with diabetes (*N* = 3673)				
Q 1 ≤ 91 mg/dL	732	44	1 [reference]	NA
Q2 92–124 mg/dL	716	67	1.19 (0.81–1.75)	0.389
Q3 125–166 mg/dL	741	75	1.14 (0.77–1.67)	0.516
Q4 167–234 mg/dL	749	87	1.24 (0.85–1.82)	0.266
Q5 ≥ 235 mg/dL	735	117	1.68 (1.15–2.45)	0.007

Abbreviations: *CI* confidence interval, *HR* hazard ratio, *NA* not applicable, *No.* number, *Q* quintile

^a^Adjusted for age, sex, ethnicity, obesity, poverty-income ratio, education, physical activity, alcohol consumption, smoking status, survey period, hypercholesterolemia, hypertension, diabetes, and family history of diabetes

### Sensitivity analyses

Sensitivity analyses showed that the association of triglyceride with diabetes mortality was attenuated but remained significant after further adjustment for baseline plasma glucose (Supporting Information Table S1), or HDL and LDL cholesterol (Supporting Information Table S2). Higher triglyceride was associated with higher serum C-reactive protein (Supporting Information Table S3), and further adjustment for serum C-reactive protein did not materially change the association between fasting triglyceride and diabetes mortality (Supporting Information Table S4). The significance of the results remained after further adjustment for use of lipid-lowering drugs (Supporting Information Table S5), or when 2-h post-load glucose of ≥200 mg/dL was considered as another criterium for diabetes diagnosis (Supporting Information Tables S6–7), or when diabetes mortality was defined as diabetes being listed as the leading cause of death (Supporting Information Table S8).

## Discussion

Using a representative cohort of US adults, this study, for the first time, revealed that higher fasting triglyceride at baseline was associated with a higher risk of diabetes mortality. Such an association was also presented in sub-cohorts of participants with or without diabetes.

The finding that higher circulating triglyceride predicted higher diabetes mortality is supported by the following findings of the current study: 1) higher triglyceride was associated with higher plasma glucose, blood HbA_1c_, serum insulin, and HOMA for insulin resistance; 2) higher triglyceride was associated with higher C-reactive protein, and the latter is an inflammatory marker and an independent predictor for diabetes [27]; and 3) higher triglyceride was associated with a higher risk of diabetes diagnosis.

The mechanism(s) underlying the association between higher triglyceride and higher diabetes mortality is not clear. One possible mechanism could be due to triglyceride-induced inflammation [28] which was supported by the observation of the current study that higher triglyceride was associated with higher levels of C-reactive protein in the circulation. However, the sensitivity analyses showed that further adjustment for C-reactive protein did not attenuate the association between triglyceride and diabetes mortality, suggesting that the association between higher triglyceride and higher diabetes mortality may not be mediated by C-reactive protein (or inflammation). Another possible mechanism could be that the change in triglyceride may be co-presented with changes in HDL and LDL cholesterol [29]. Sensitivity analyses showed that further adjustment for HDL and LDL cholesterol did attenuate but did not abolish the association between triglyceride and diabetes mortality. Baseline glucose was a strong predictor of future diabetes [27]. Future adjustment for baseline plasma glucose did attenuate but did not abolish the association between triglyceride and diabetes mortality. All these results suggest that circulating triglyceride was an independent risk factor for future diabetes mortality, and the association may be partially mediated by plasma glucose, and HDL and LDL cholesterol.

Epidemiological [2, 3] and longitudinal cohort studies [4–6, 30, 31] showed that higher triglyceride may be a cause of new-onset of diabetes. However, this is not without controversy, as genetic studies provided mixed results. For example, triglyceride-increasing alleles have been reported to be either not associated with [32], or positively associated with [33], or inversely associated with diabetes [6, 34, 35]. One of the genetic studies [6] revealed inconsistency between genetic and non-genetic results from the same cohort: triglyceride-increasing alleles were associated with lower new-onset of diabetes, whereas higher baseline circulating levels of triglyceride were associated with higher new-onset of the disease. The reason underlying the observed inconsistency [6] is unknown; it might be due to the selection of genetic alleles or due to that some of the selected genetic alleles have other functions beyond regulating circulating triglyceride.

In people without diabetes at baseline, higher fasting triglyceride predicted impaired glucose tolerance [36], impaired fasting glucose (IFG), and new-onset of diabetes [4–6, 29, 30]. The results of the current study showed that, in participants without diabetes at baseline, higher fasting triglyceride predicted diabetes mortality, supporting the notion that higher triglyceride may be a cause rather than an effect of diabetes.

It has been reported that higher triglyceride was associated with higher all-cause mortality [37, 38]. The current study suggested that the positive association between triglyceride and all-cause mortality may be mediated, at least in part, by higher diabetes mortality. Diabetes can lead to increased risks for cardiovascular disease including heart failure, and glycemic control drugs including sodium-glucose transporter 2 (SGLT2) inhibitors have substantially improved the management of patients with diabetes and reduced patients’ cardiovascular complications [39]. Higher fasting triglyceride has been shown to be associated with higher cardiovascular disease mortality [40]. Therefore, reduced cardiovascular disease mortality might be involved in the reduced all-cause mortality associated with lower triglyceride.

### Comparisons with other studies and what does the current work add to the existing knowledge

Although it has been widely reported that higher triglyceride is associated with diabetes prevalence and incidence [2–6, 30, 31], one important piece of information is missing from the literature, i.e., whether higher triglyceride is associated with higher diabetes mortality. The current study filled this knowledge gap. Diabetes mortality could be defined as diabetes being listed as an underlying cause of death or as the leading cause of death. In either case, higher triglyceride was found to be associated with higher diabetes mortality in the general US population.

### Strengths and limitations

A strength of this study is its large sample size (*N* = 26,582). This study has several other strengths, such as a prospective study design, the use of a nationally representative sample of US adults with or without diabetes, and adjustment for a large number of confounding factors. This study also has a number of limitations. First, triglyceride was only measured at one time point, which may result in misclassification. Nevertheless, this misclassification tends to result in an underestimate rather than an overestimate of risk in epidemiological analysis due to the effect of regression dilution bias [41]. Second, mortality outcomes were ascertained by linkage to the National Death Index (NDI) records with a probabilistic match, which may also lead to misclassification. However, the Special Projects Branch from the National Center for Health Statistics employed a matching methodology offered by the NDI to determine the best match [42] and a prior validation study showed that the matching method had high accuracy (98.5%) [43].

## Conclusion

**Fig. 1:**
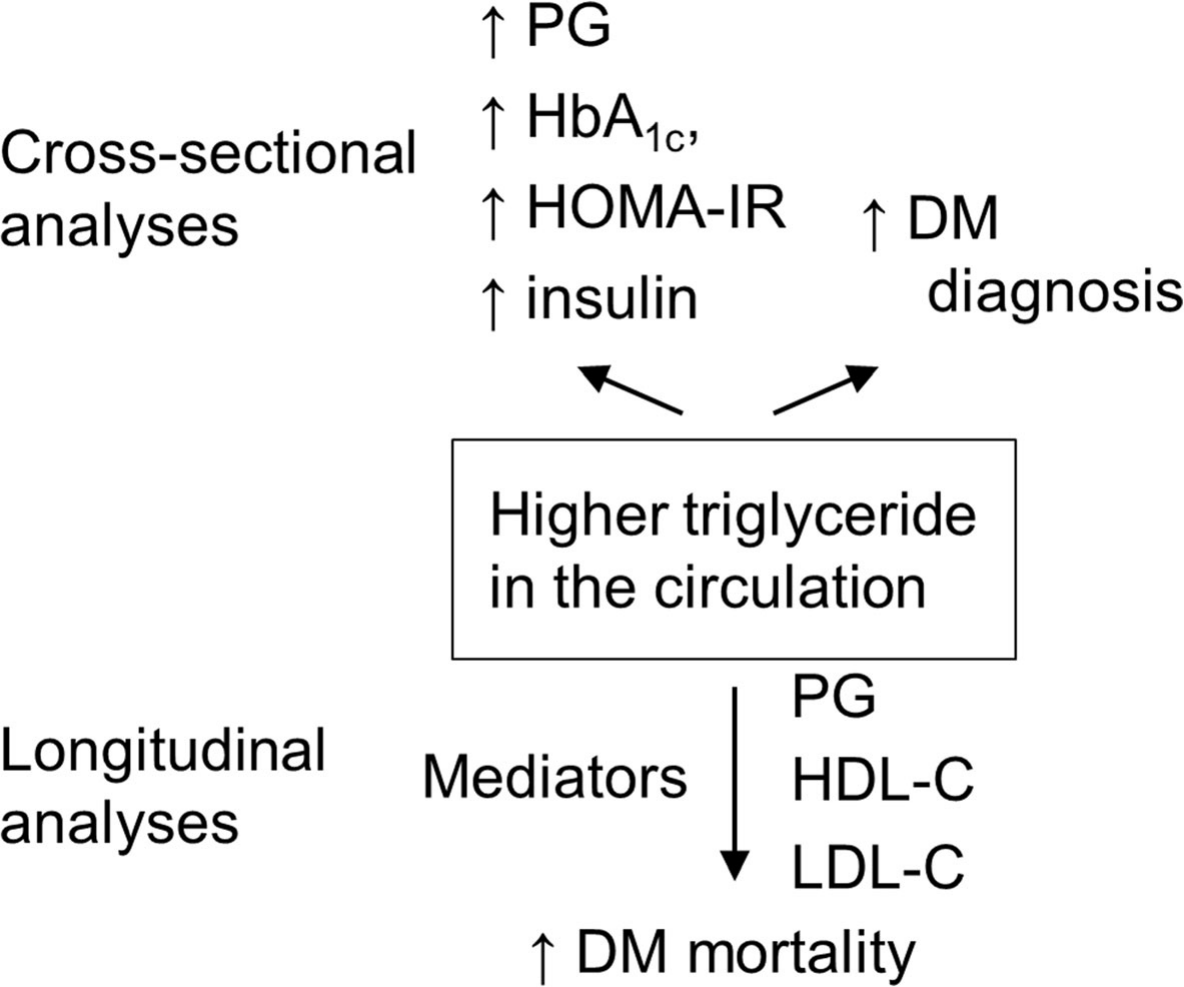
Summary of the study. In the cross-sectional analyses, higher triglyceride was associated with higher levels of diabetes markers and higher risks for diabetes diagnosis. In the longitudinal analyses, higher triglyceride predicted higher diabetes mortality, which might be partially mediated by PG, HDL cholesterol, and LDL cholesterol, but not by C-reactive protein. ↑, increased; DM, diabetes; HbA1c, glycated hemoglobin; HDL-C, high-density lipoprotein cholesterol; HOMA-IR, homeostatic model assessment for insulin resistance; LDL-C, low-density lipoprotein cholesterol; PG, plasma glucose

This study demonstrated that higher fasting triglyceride was associated with a higher risk of diabetes diagnosis and diabetes mortality (Fig. 1). Triglyceride may need to be routinely monitored in clinic for the risk assessment for diabetes. In addition, triglyceride may be a therapeutic target for the management of diabetes, and clinical trials on the effectiveness of lowering triglyceride to prevent diabetes mortality are needed.

## Supplementary Information

**Additional file 1.** Supporting information. Tables S1-S8.

## Data Availability

The datasets supporting the conclusions of this article are publicly available on the NHANES website, https://www.cdc.gov/nchs/nhanes/index.htm.

## Abbreviations

CI: Confidence interval
CRP: C-reactive protein
HbA1c: Glycated hemoglobin
HDL: High-density lipoprotein
HOMA: Homeostatic model assessment
HOMA-IR: Homeostatic model assessment for insulin resistance
HR: Hazard ratio
ICD: International Classification of Diseases
LDL: Low-density lipoprotein
NA: Not applicable
NDI: National Death Index
NHANES: National Health and Nutrition Examination Survey
No.: Number
PG: Plasma glucose
Q: Quintile
SD: Standard deviation
UCOD: Underlying Cause of Death
