# Supplementary material for: Higher fasting triglyceride predicts higher risks of diabetes mortality in US adults
Source: Lipids Health Dis. 2021 Dec 20;20:181. doi: 10.1186/s12944-021-01614-6 (PMC8686260; doi:10.1186/s12944-021-01614-6)
Supplement: Supplementary file 1 — Additional file 1. Supporting information. Tables S1-S8. [file 12944_2021_1614_MOESM1_ESM.docx]

**Supporting Information**

**Table S1** Natural log-transformed triglyceride and risk for diabetes mortality in 26,582 adults before and after further adjustment for baseline plasma glucose

|  | Hazard ratio | 95% CI | *P* value |
| --- | --- | --- | --- |
| Model 1 | 1.40 | 1.20-1.64 | <0.001 |
| Model 2 | 1.26 | 1.08-1.48 | 0.004 |

Abbreviations: CI, confidence interval.

Model 1: adjusted for age, sex, ethnicity, obesity, poverty-income ratio, education, physical activity, alcohol consumption, smoking status, survey period, hypercholesterolemia, hypertension, diabetes, and family history of diabetes; Model 2: adjusted for all the factors in Model 1 plus natural log-transformed baseline plasma glucose.

**Table S2** Natural log-transformed triglyceride and risk for diabetes mortality in 23,498 adults ^a^ before and after further adjustment for HDL and LDL cholesterol

|  | Hazard ratio | 95% CI | *P* value |
| --- | --- | --- | --- |
| Model 1 | 1.52 | 1.23-1.88 | <0.001 |
| Model 2 | 1.34 | 1.06-1.70 | 0.015 |

Abbreviations: CI, confidence interval; HDL, high-density lipoprotein; LDL, low-density lipoprotein.

^a^ A total of 3,084 people who did not have HDL or LDL cholesterol data were excluded and the analysis included the remaining 23,498 participants.

Model 1: adjusted for age, sex, ethnicity, obesity, poverty-income ratio, education, physical activity, alcohol consumption, smoking status, survey period, hypercholesterolemia, hypertension, diabetes, and family history of diabetes; Model 2: adjusted for all the factors in Model 1 plus natural log-transformed HDL and LDL cholesterol.

**Table S3** Association of fasting triglyceride (independent variable) with C-reactive protein (dependent variables) in 21,959 adults ^a^

|  | Model 1 | | Model 2 | | Model 3 | | Model 4 | |
| --- | --- | --- | --- | --- | --- | --- | --- | --- |
|  | β | *P* value | β | *P* value | β | *P* value | β | *P* value |
| All participants (N=26,582) | 0.183 | <0.001 | 0.198 | <0.001 | 0.101 | <0.001 | 0.100 | <0.001 |
| Participants without diabetes (N=22,909) | 0.171 | <0.001 | 0.188 | <0.001 | 0.093 | <0.001 | 0.094 | <0.001 |
| Participants with diabetes (N=3,673) | 0.126 | <0.001 | 0.154 | <0.001 | 0.101 | <0.001 | 0.115 | <0.001 |

^a^ Triglyceride and C-reactive protein were natural log transformed. A total of 4,623 people who did not have serum C-reactive protein data were excluded and the analysis included the remaining 21,959 participants.

Model 1: unadjusted; Model 2: adjusted for age, sex, and ethnicity; Model 3: adjusted for all the factors in Model 2 plus obesity, poverty-income ratio, education, physical activity, alcohol consumption, smoking status, and survey period; Model 4: adjusted for all the factors in Model 3 plus hypercholesterolemia, hypertension, diabetes, and family history of diabetes.

**Table S4** Fasting triglyceride and risk for diabetes mortality in 21,959 adults before and after further adjustment for C-reactive protein ^a^

|  | Hazard ratio | 95% CI | *P* value |
| --- | --- | --- | --- |
| Model 1 | 1.39 | 1.19-1.63 | <0.001 |
| Model 2 | 1.36 | 1.16-1.60 | <0.001 |

Abbreviations: CI, confidence interval.

^a^ Triglyceride was natural log transformed. A total of 4,623 people who did not have serum C-reactive protein data were excluded and the analysis included the remaining 21,959 participants.

Model 1: adjusted for age, sex, ethnicity, obesity, poverty-income ratio, education, physical activity, alcohol consumption, smoking status, survey period, hypercholesterolemia, hypertension, diabetes, and family history of diabetes; Model 2: adjusted for all the factors in Model 1 plus natural log-transformed C-reactive protein.

**Table S5** Natural log-transformed triglyceride and risk for diabetes mortality in 26,582 adults before and after further adjustment for use of lipid-lowering drugs

|  | Hazard ratio | 95% CI | *P* value |
| --- | --- | --- | --- |
| Model 1 | 1.40 | 1.20-1.64 | <0.001 |
| Model 2 | 1.42 | 1.21-1.65 | <0.001 |

Abbreviations: CI, confidence interval.

Model 1: adjusted for age, sex, ethnicity, obesity, poverty-income ratio, education, physical activity, alcohol consumption, smoking status, survey period, hypercholesterolemia, hypertension, diabetes, and family history of diabetes; Model 2: adjusted for all the factors in Model 1 plus use of lipid-lowering drugs.

**Table S6** Sensitivity analysis of association of triglyceride (independent variable)^a^ with diabetes markers (dependent variables) ^a^ in 26,582 adults when 2-hour post-load glucose of ≥200 mg/dL was considered as another criterium for diabetes diagnosis

|  | Model 1 | | Model 2 | | Model 3 | | Model 4 | |
| --- | --- | --- | --- | --- | --- | --- | --- | --- |
|  | β | *P* value | β | *P* value | β | *P* value | β | *P* value |
| All participants (N = 26,582) | | | | | | | | |
| Plasma glucose | 0.275 | <0.001 | 0.226 | < 0.001 | 0.193 | < 0.001 | 0.116 | < 0.001 |
| Blood HbA_1c_ | 0.223 | <0.001 | 0.192 | < 0.001 | 0.159 | < 0.001 | 0.076 | < 0.001 |
| Serum insulin | 0.367 | <0.001 | 0.395 | < 0.001 | 0.268 | < 0.001 | 0.257 | < 0.001 |
| HOMA-IR | 0.401 | <0.001 | 0.411 | < 0.001 | 0.290 | < 0.001 | 0.259 | < 0.001 |
| Participants without diabetes ^b^ (N = 22,588) | | | | | | | | |
| Plasma glucose | 0.198 | <0.001 | 0.118 | < 0.001 | 0.075 | < 0.001 | 0.073 | < 0.001 |
| Blood HbA_1c_ | 0.116 | <0.001 | 0.084 | < 0.001 | 0.048 | < 0.001 | 0.034 | < 0.001 |
| Serum insulin | 0.356 | <0.001 | 0.392 | < 0.001 | 0.266 | < 0.001 | 0.267 | < 0.001 |
| HOMA-IR | 0.364 | <0.001 | 0.385 | < 0.001 | 0.260 | < 0.001 | 0.261 | < 0.001 |
| Participants with diabetes ^b^ (N = 3,994) | | | | | | | | |
| Plasma glucose | 0.285 | <0.001 | 0.286 | < 0.001 | 0.280 | < 0.001 | 0.282 | < 0.001 |
| Blood HbA_1c_ | 0.204 | <0.001 | 0.222 | < 0.001 | 0.209 | < 0.001 | 0.197 | < 0.001 |
| Serum insulin | 0.263 | <0.001 | 0.263 | < 0.001 | 0.215 | < 0.001 | 0.221 | < 0.001 |
| HOMA-IR | 0.350 | <0.001 | 0.351 | < 0.001 | 0.306 | < 0.001 | 0.312 | < 0.001 |

Abbreviations: HbA_1c_, glycated hemoglobin; HOMA-IR, homeostatic model assessment for insulin resistance.

^a^ Triglyceride, plasma glucose, blood HbA_1c_, serum insulin, and HOMA-IR, were natural log-transformed.

^b^ Diabetes was defined as fasting plasma glucose ≥ 126 mg/dL, or glycated hemoglobin (HbA_1c_) ≥ 6.5%, **or glucose at 2 hours after 75-g glucose load (oral glucose tolerance test) ≥200 mg/d**L, or taking hypoglycemic drugs, or self-reported diagnosis [14]. By this definition, 321 participants who were previously classified as without diabetes when 2-hour post-load glucose was not considered were classified as with diabetes.

Model 1: unadjusted; Model 2: adjusted for age, sex, and ethnicity; Model 3: adjusted for all the factors in Model 2 plus obesity, poverty-income ratio, education, physical activity, alcohol consumption, smoking status, and survey period; Model 4: adjusted for all the factors in Model 3 plus hypercholesterolemia, hypertension, diabetes, and family history of diabetes.

**Table S7** Sensitivity analysis of natural log-transformed triglyceride and risk for diabetes mortality among 26,582 adults, after considering 2-hour post-load glucose of ≥200 mg/dL as another criterium for diabetes diagnosis

|  | All participants  (N = 26,582) | | | Participants without diabetes ^a^ (N = 22,588) | | | Participants with diabetes ^a^ (N = 3,994) | | |
| --- | --- | --- | --- | --- | --- | --- | --- | --- | --- |
|  | HR | 95% CI | *P* value | HR | 95% CI | *P* value | HR | 95% CI | *P* value |
| Model 1 | 2.62 | 2.33-2.94 | <0.001 | 2.23 | 1.78-2.80 | <0.001 | 1.34 | 1.15-1.56 | <0.001 |
| Model 2 | 2.35 | 2.05-2.68 | <0.001 | 1.77 | 1.37-2.29 | <0.001 | 1.51 | 1.27-1.79 | <0.001 |
| Model 3 | 2.12 | 1.84-2.45 | <0.001 | 1.50 | 1.14-1.98 | 0.004 | 1.41 | 1.18-1.69 | <0.001 |
| Model 4 | 1.40 | 1.20-1.64 | <0.001 | 1.43 | 1.07-1.91 | 0.015 | 1.33 | 1.11-1.60 | 0.003 |

Abbreviations: CI, confidence interval; HR, hazard ratio.

^a^ Diabetes was defined as fasting plasma glucose ≥ 126 mg/dL, or glycated hemoglobin (HbA_1c_) ≥ 6.5%, **or** **glucose at 2 hours after 75-g glucose load (oral glucose tolerance test) ≥200 mg/d**L, or taking hypoglycemic drugs, or self-reported diagnosis. By this definition, 321 participants who were previously classified as without diabetes when 2-hour post-load glucose was not considered were classified as with diabetes.

Model 1: unadjusted; Model 2: adjusted for age, sex, and ethnicity; Model 3: adjusted for all the factors in Model 2 plus obesity, poverty-income ratio, education, physical activity, alcohol consumption, smoking status, and survey period; Model 4: adjusted for all the factors in Model 3 plus hypercholesterolemia, hypertension, diabetes, and family history of diabetes.

**Table S8** Sensitivity analysis of natural log-transformed triglyceride and risk for diabetes mortality ^a^ among 17,185 adults

|  | HR | 95% CI | *P* value |
| --- | --- | --- | --- |
| Model 1 | 2.98 | 2.37-3.76 | <0.001 |
| Model 2 | 2.66 | 2.04-3.48 | <0.001 |
| Model 3 | 2.43 | 1.82-3.24 | <0.001 |
| Model 4 | 1.64 | 1.20-2.24 | 0.002 |

Abbreviations: CI, confidence interval; HR, hazard ratio.

^a^ Diabetes mortality was defined as diabetes being listed as the leading cause of death. The data on diabetes being listed as the leading cause of death were only available for participants in NHANES 1988-2006.. In this 1988-2006 sub-cohort of 17,185 participants, 130 deaths were recorded with diabetes being the leading cause of death.

Model 1: unadjusted; Model 2: adjusted for age, sex, and ethnicity; Model 3: adjusted for all the factors in Model 2 plus obesity, poverty-income ratio, education, physical activity, alcohol consumption, smoking status, and survey period; Model 4: adjusted for all the factors in Model 3 plus hypercholesterolemia, hypertension, diabetes, and family history of diabetes.
